# Polymorphism in carbohydrate self-assembly at surfaces: STM imaging and theoretical modelling of trehalose on Cu(100)†

**DOI:** 10.1039/c9ra06764g

**Published:** 2019-11-04

**Authors:** Sabine Abb, Nathalie Tarrat, Juan Cortés, Bohdan Andriyevsky, Ludger Harnau, J. Christian Schön, Stephan Rauschenbach, Klaus Kern

**Affiliations:** Max Planck Institute for Solid State Research Heisenbergstr. 1 70569 Stuttgart Germany s.abb@fkf.mpg.de; CEMES, Université de Toulouse, CNRS 29 Rue Jeanne Marvig 31055 Toulouse France; LAAS-CNRS, Université de Toulouse, CNRS Toulouse France; Koszalin University of Technology Śniadeckich Str. 2 75-453 Koszalin Poland; Bernhäuser Str. 75 70771 Leinfelden-Echterdingen Germany; Department of Chemistry, University of Oxford Mansfield Road Oxford OX1 3TA UK; Institut de Physique, Ecole Polytechnique Fédérale de Lausanne 1015 Lausanne Switzerland

## Abstract

Saccharides, also commonly known as carbohydrates, are ubiquitous biomolecules, but little is known about their interaction with surfaces. Soft-landing electrospray ion beam deposition in conjunction with high-resolution imaging by scanning tunneling microscopy now provides access to the molecular details of the surface assembly of this important class of bio-molecules. Among carbohydrates, the disaccharide trehalose is outstanding as it enables strong anhydrobiotic effects in biosystems. This ability is closely related to the observed polymorphism. In this work, we explore the self-assembly of trehalose on the Cu(100) surface. Molecular imaging reveals the details of the assembly properties in this reduced symmetry environment. Already at room temperature, we observe a variety of self-assembled motifs, in contrast to other disaccharides like *e.g.* sucrose. Using a multistage modeling approach, we rationalize the conformation of trehalose on the copper surface as well as the intermolecular interactions and the self-assembly behavior.

## Abstract 2

We rationalize the experimentally observed variety of trehalose assemblies on Cu(100) by modeling based on STM images and global optimization.

## Graphical abstract

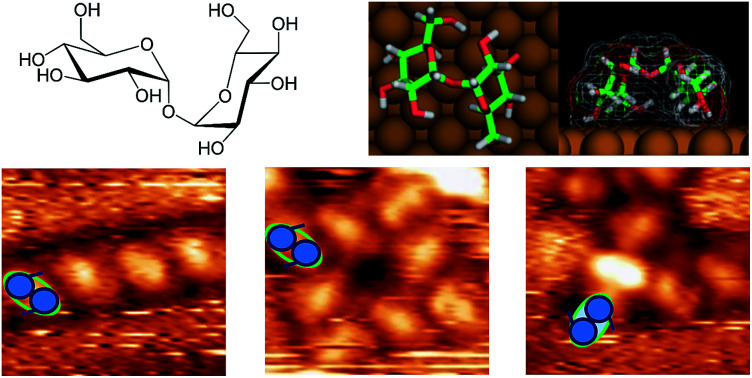

## Introduction

**Fig. 1:**
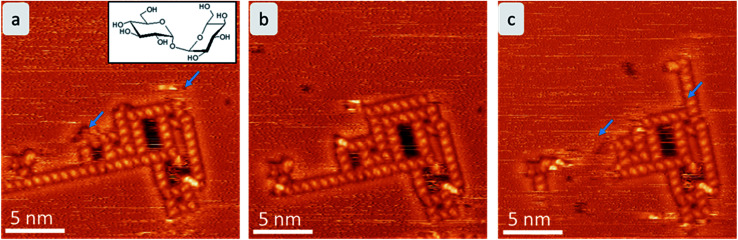
Time evolution of trehalose assemblies at room temperature. Each frame takes 6 minutes. The areas that change in consecutive images are marked with a blue arrow. Here, mostly the linear assembly motif (motif A) is affected by the fluctuations.

Trehalose (α-d-glucopyranosyl-(1→1)-α-d-glucopyranoside; see inset Fig. 1) is a non-reducing disaccharide, which is known for its polymorphism and its anhydrobiotic properties.^1–3^ It crystallizes in anhydrous form in at least two modifications, for which X-ray data exist, in a further metastable phase whose existence is only deduced from differential scanning calorimetry (DSC) measurements, and finally in an amorphous glassy state.^1,2,4^ Furthermore, the formation of trehalose agglomerates such as clusters, chains, layers, and structures containing channels or holes^1,5^ on the surfaces/interfaces of the biomolecular systems appears to constitute an important feature for its biological function.

Relating the molecular structure of trehalose to its conformations is extremely challenging. NMR,^6,7^ X-ray^1^ and neutron scattering^8,9^ measurements have been performed to probe the trehalose–water interactions, in particular, which were complemented by numerical simulations.^1,5,10,11^ Beyond that, the conformations and assembly motifs of saccharides in environments of reduced symmetry, *e.g.* on surfaces and in contact with few/other molecules, remain essentially unknown.

A suitable technique to study the structure and conformation of adsorbed (bio)molecules is high-resolution scanning probe microscopy enabled by gentle and chemically selective electrospray ion beam deposition (ES-IBD),^12,13^ because it allows the direct imaging of nonvolatile molecules with sub-molecular resolution.^14–16^ We applied this methodology in a recent proof-of-concept study observing periodic assemblies of sucrose molecules on a Cu(100) surface.^17^ We were able to identify monosaccharide subunits and to gain further atomic details by exploration of the conformational space of the molecule–surface system through a multi-scale theoretical modeling approach.^18^

In the present study, we explore the self-assembly and conformations of trehalose adsorbed on a Cu(100) surface. Despite the significant difference between the metal surface in UHV and the surface of a protein or lipid bilayer, this exploratory study exemplifies the aggregation motifs of trehalose on extended surfaces. Our approach employing a combination of direct imaging of the trehalose molecule by scanning tunneling microscopy (STM) and multi-stage modelling of the observed structures allows us to obtain the conformations of individual trehalose molecules on the surface, and to study structure and binding in observed self-assembled patterns.

## Results

### STM observation of trehalose on Cu(100)

After mass-selected deposition of trehalose on Cu(100), ordered anisotropic structures are observed by STM at room temperature. In the time series (see Fig. 1), fluctuations and rearrangements of these assemblies on the time scale of an image (2–6 min) indicate that the molecules are mobile on the surface. The mobility of the molecules on the surface is also supported by the streaks observed in the STM images.

**Fig. 2:**
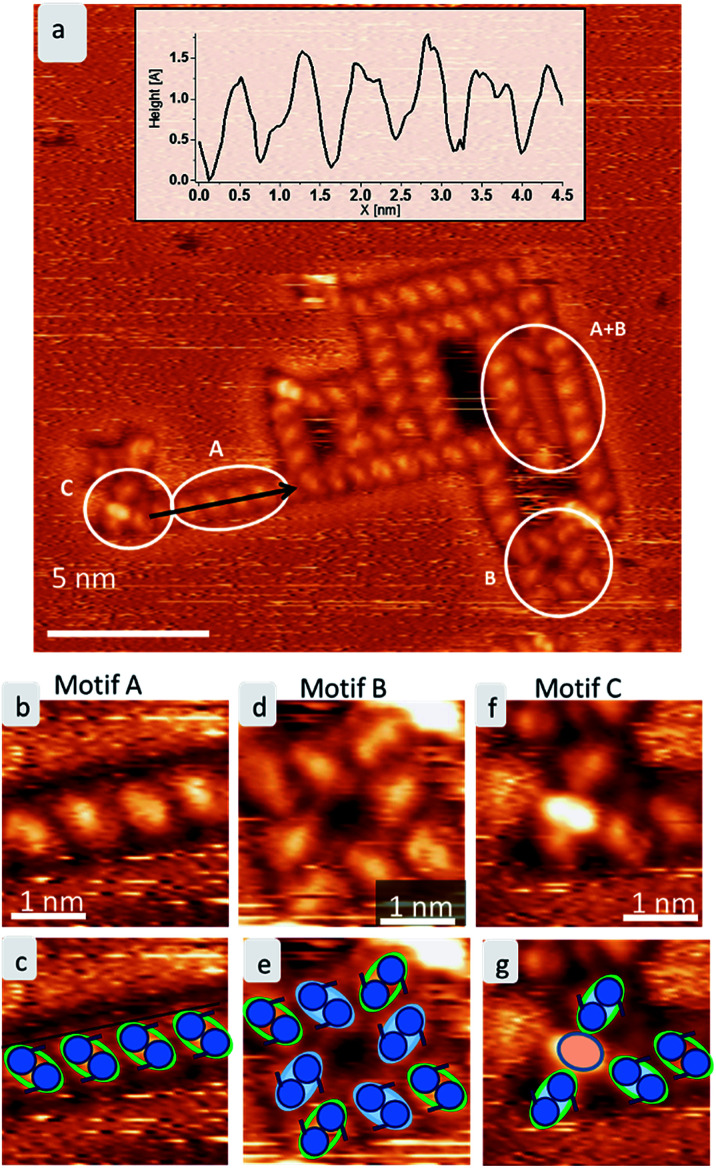
The three ordered motifs A–C of trehalose on Cu(100). (a) STM image showing the assembly of trehalose on Cu(100) at room temperature. The three different motifs A–C are marked. A line profile is shown in the inset. (b) Magnification of motif A depicting a line of skewed molecules. (c) A sketch of motif A. The green lobes correspond to single trehalose molecules, the two blue circles indicate the two saccharide subunits. (d) Zoom into motif B depicting the squared arrangement of 8 trehalose molecules. (e) Sketch of motif B. (f) Zoom into motif C. (g) Sketch of motif C.

In all assemblies, the smallest, clearly resolved features are 0.9 ± 0.1 nm in length and 0.6 ± 0.1 nm in width. These dimensions agree well with the expected size of a single molecule. Thus, each feature is attributed to a single trehalose molecule. Within some molecular features, substructure is visible as intensity/height variations. However, this substructure varies between molecules and thus does not allow to resolve the individual monosaccharide building units. Three different assembly motifs are identified and marked A–C (Fig. 2). All motifs are chiral with only one enantiomer found on the surface. Motif A is an anisotropic assembly formed by parallel aggregation of molecules along their long axis as shown in Fig. 2b. The spacing along the line of molecules of 0.8 ± 0.1 nm is depicted in the line scan in Fig. 2a. Within the lines, the molecules are rotated by 46° ± 6° anticlockwise with respect to the orientation of the linear assembly as a whole. The observed orientations of the line arrays are rotated by 90° with respect to one another, reminiscent of the fourfold symmetry of the Cu(100) surface.

Motif B is an assembly consisting of eight molecules in a square arrangement with *C*_4_ symmetry, as seen in Fig. 2d. The dimensions of this assembly are 1.5 ± 0.2 nm in width and 2.3 ± 0.2 nm along the diagonal. Four molecules (highlighted in blue in the cartoon in Fig. 2e) form a windmill-like motif, creating a pore in the center. The remaining four molecules (green outline in Fig. 2e) are located at the corners of the assembly, aligning with the neighboring molecules. These molecules are oriented on the surface in the same way as in motif A.

Due to the agreement in orientation of the molecules within motif A and motif B, assemblies built from one of the motifs can seamlessly transition into an assembly based on the other motif. In addition, different combinations of these two motifs are observed. Integrating motif A into motif B yields, *e.g.*, large hollow rectangles by elongating one side of the square due to the linear motif A (see Fig. 2a indicated by A + B). It is frequently observed that the space inside these rectangles is filled with molecules. However, these are usually mobile and can only be imaged as unstructured features.

A third motif, motif C (Fig. 2f), is observed terminating some of the linear assemblies. It is characterized by a bright central feature – about the size of one molecule, which consists of one or two bright spots, plus several (up to four) molecules. This motif is clearly smaller and more densely packed than the windmill arrangement in motif B. The central feature is surrounded by up to four molecules, which have the same orientation as the corner molecules in motif B. Therefore, this motif can also connect to motif A.

The same motifs A–C are observed also at low temperature (room temperature deposition), as shown in ESI Fig. SI-1.† Interestingly, the assemblies are larger and more regular at low temperature forming a porous network of a combination of all motifs A–C. These networks most likely form during the cooling down process and can therefore be understood as first step towards crystallization of larger regular structures. Moreover, single molecules and aggregates are observable at low temperature, demonstrating that most of the molecules were mobile at room temperature.

### Models of trehalose adsorption on Cu(100)

In order to shed light on the molecular conformation and interactions in the experimentally observed assemblies, we modelled the structures at the atomic level. For modelling, we have to take into account the flexibility of the individual molecule and its possible different adsorption conformations as well as the large surface area that the assembly can occupy. Thus, we employed a multi-stage procedure to accommodate all these requirements, as often done in such a case.^17–20^

In a first step, we used the Iterative Global exploration and LOcal Optimization (IGLOO) scheme to find suitable conformations of the single disaccharide on the surface (see ESI† for details). Using dispersion-corrected DFT, these single molecule conformations were locally optimized, where we relaxed the constraints on bond lengths and bond angles that had been imposed for the global exploration. The minimized conformations exhibit improved molecule–surface interactions as well as larger intramolecular interactions due to stronger hydrogen bonds. In a last step, we employed the single molecule conformations to obtain models of the assemblies according to constraints defined from the STM images, such as dimensions and symmetry, including chirality. We took several precautions to ensure that the optimized single molecule conformation can indeed be used as a building block for designing the assemblies (see ESI†).

**Fig. 3:**
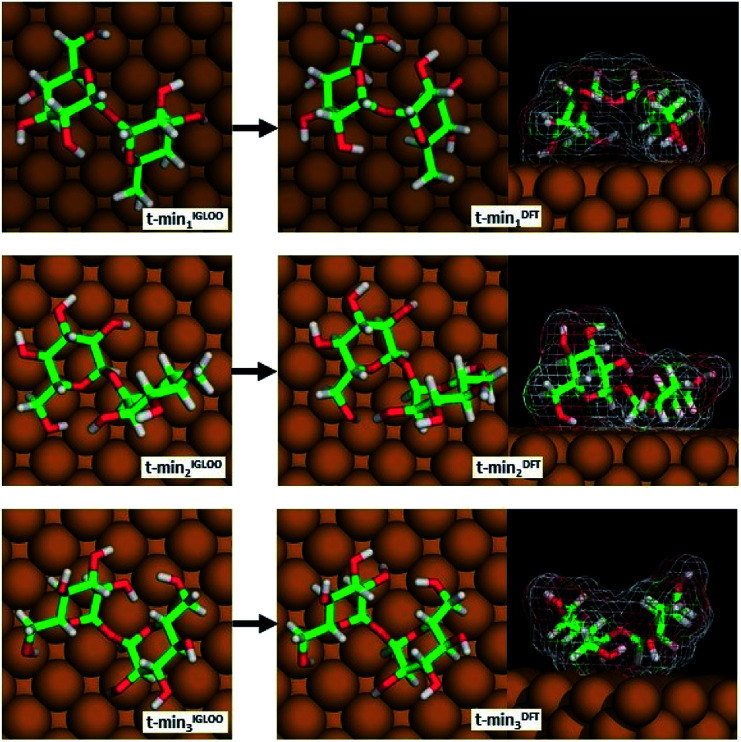
The three lowest-energy conformations of trehalose on Cu(100) obtained by the IGLOO algorithm (left image) and after DFT minimization (right image).

From among the many local minima found by the IGLOO method for the single trehalose molecule on the Cu(100) surface, three low-energy conformations belonging to different minimum basins with distinct conformations were chosen for further analysis (labelled t-min_1_^IGLOO^, t-min_2_^IGLOO^ and t-min_3_^IGLOO^ in the following; see Fig. 3; the order of the minima was chosen according to their energies after the subsequent DFT minimization). The energy differences with respect to t-min_2_^IGLOO^ (the one lowest in energy on empirical potential level) are approximately 6.3 kJ mol^−1^ (65 meV) for t-min_3_^IGLOO^ and 23.0 kJ mol^−1^ (238 meV) for t-min_1_^IGLOO^. These small differences suggest that a single trehalose molecule can adopt these different conformations on the Cu(100) surface.

For t-min_2_^IGLOO^, the change from IGLOO to DFT optimized configuration includes a slight shift on the surface as well as small changes in some of the dihedral angles involving hydroxyl groups close to the surface. As seen in the side view (right panel in Fig. 3), one glucose ring is standing nearly upright pointing towards the surface with the ring oxygen and O6, while the other ring is nearly flat on the surface. This yields a topography in which one glucose unit of the molecule is higher than the other one by 2.4 Å. For t-min_3_^IGLOO^, the orientation on the surface does not change extensively and only minor changes in bond angles and bond lengths are observable. This molecular conformation yields a bowl-like structure on the surface, in which the ends of the molecule stand up. Similar to t-min_2_^IGLOO^, the DFT optimization of t-min_1_^IGLOO^ induces a reorientation on the surface and small changes in the conformation of the molecule. As shown in the side view (Fig. 3), the molecule is bent in such a way that both rings point to the surface with the C5 atom, yielding a bridge-like structure in which the center part, such as the bridging oxygen, is lifted away from the surface.

These changes have a direct effect on the energy ranking, which is modified with respect to the initial ranking. After minimizing t-min_1,2,3_^IGLOO^ on DFT level, we obtain the structures t-min_1,2,3_^DFT^, respectively. The lowest-energy conformation at DFT level is t-min_1_^DFT^, the middle conformation is t-min_2_^DFT^, and the energetically highest one is t-min_3_^DFT^. The potential energy differences with respect to t-min_1_^DFT^ are approximately 10.5 kJ mol^−1^ (109 meV) for t-min_2_^DFT^ and 21.3 kJ mol^−1^ (221 meV) for t-min_3_^DFT^, respectively. Note that these differences are of the same order of magnitude as those obtained with the empirical potential.

These three lowest-energy structures were subsequently employed to obtain models for the observed assemblies. Since we can only speculate about the nature of the central high intensity in motif C, we will not present an atomic model for this motif.

**Fig. 4:**
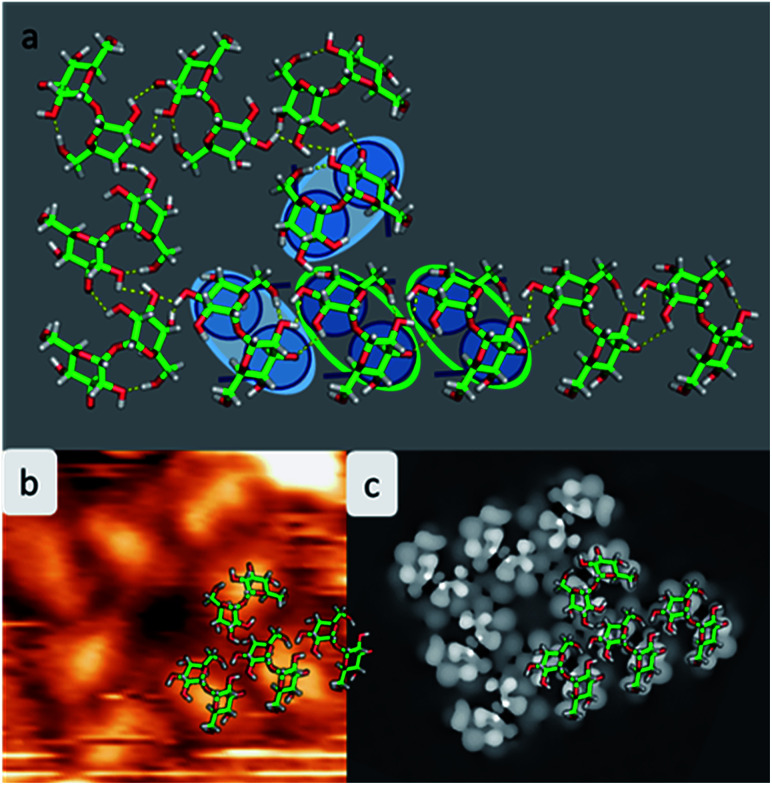
Model of the assembly patterns for trehalose. (a) Atomistic model of the assembly motif A merged with motif B with hydrogen bonds indicated in yellow. The copper atoms are not shown to avoid overloading the picture. For clarification a corner of the assembly is underlaid with the cartoon. (b) Measured STM images with one quarter of the structure overlaid with the molecular structure of the pattern. (c) Computed STM image showing the highest contrast in the center of the molecule.

Experimental restrictions, such as the chirality and the distances between the features extracted from the STM data, drastically limit the number of possible structures. A discussion concerning all possible assembly patterns is given in the ESI (Fig. SI-3†). Fig. 4 depicts the best-fitting model of trehalose assemblies of motif A and motif B, which was achieved by using t-min_1_^DFT^.

As shown in Fig. 4a, the molecules assemble in parallel to form motif A, yielding four intermolecular hydrogen bonds. Furthermore, the periodicity of 7.6 Å, which is imposed to be commensurate with the substrate along the [110] direction, as well as the orientation of the molecules with respect to the direction of the alignment is in good agreement with the STM data.

With this procedure, motif B can also be formed, taking the restrictions deduced from the STM data into account. Here, the molecules form a motif with 4-fold symmetry, which is chiral due to the rotation of the molecules with respect to the surface main direction. In this assembly pattern, the inner windmill structure (blue outline in Fig. 2) is stabilized by four intermolecular hydrogen bonds, while the molecules at the corners exhibit up to six intermolecular hydrogen bonds. Note that those molecules are also the starting point for motif A. As observed in the STM images, the linear assembly of motif A and the octamer of motif B can smoothly merge.

The good agreement of the STM image generated using the HIVE program^21^ with the experimental data validates the model, as shown in Fig. 4b and c. The simulated STM image shows a combined pattern of motif A and B consisting of 9 trehalose molecules (8 + 1) in the t-min_1_^DFT^ conformation. Due to the (over-turned) bowl-like conformation of t-min_1_^DFT^ on the surface, the highest intensity features are located on both monosaccharide units close to the oxygen bridge. Taking into account the tip convolution, the single units are not clearly distinguishable and thus, the molecule is observed with one bright feature in the central part. This single symmetric bright feature in the STM image is only possible with the conformation t-min_1_^DFT^, because the other low energy conformations exhibit either an asymmetric conformation with one building block being higher than the other one (t-min_2_^DFT^) or exhibiting the highest topographic features at the ends of the molecule (*cf.* HIVE STM image for t-min_3_^DFT^ in ESI Fig. SI-8†).

## Discussion

Comparing the three different configurations of the trehalose molecule on Cu(100) (Fig. 3) to the three most stable low-energy minima found from a global optimization of trehalose in vacuum and to the molecular structure observed in the anhydrous crystalline modifications Tre(α) allows us to identify the influence of the surface on the molecular configuration. The overall structures on the surface, in vacuum and in the crystalline environment are similar as far as bond lengths and bond angles are concerned (see ESI Fig. SI-4, SI-5 and Table SI-1†). The main difference is in the relative orientation of the two rings, which is reflected in the dihedral angles *ψ*_H_ and *ϕ*_H_ (see ESI Fig. SI-5†). This is not surprising because our global landscape studies on the surface, and also in vacuum,^22^ have shown that the barrier against rotations around the corresponding bonds is rather small in disaccharides. This high degree of similarity supports our expectation that some features of the three-dimensional assemblies of trehalose molecules will also appear for trehalose 2D assemblies on a surface.

Additionally, we recall that anhydrous trehalose crystallizes in several different crystal structures, and exhibits at least one amorphous phase^1,2^ after a glass transition. It was also noted that the growth of the crystals occurs in a quasi-two-dimensional fashion.^1,23^ This supports the appearance of multiple stable structural motifs also in two-dimensional trehalose assemblies. Furthermore, in studies on systems containing trehalose,^1^ trehalose and water,^1,10,11^ or trehalose on a biomolecule,^5^ trehalose molecules were observed to form chains and fractal clusters,^11^ cluster aggregates,^1^ various structural blocks containing hollow sites,^1^ and clusters on surfaces of biomolecules.^5^ Analogous two-dimensional patterns are observed in the trehalose molecule assemblies on the Cu surface in this study.

An important feature of the trehalose aggregates is the lack of long-range order, leading to variations and combinations of small assembly patterns. In order to understand this, we need to address (i) the existence of several basic structural motifs of similar formation energy, including intermolecular interactions, (ii) the ability of a given motif to form periodic structures by itself on the surface (discussing the interaction with the surface and its commensurability), and, finally, (iii) the mutual compatibility of these motifs within larger aggregates of trehalose molecules and the interchange between these motifs due to mobility.

We have to keep in mind that the various structural motifs we observed do not appear to differ greatly in energy. Thus no strong thermodynamic force exists that would favor certain motifs. Nonetheless, motif B, once formed, is stable enough to avoid being broken up. In contrast, the terminal trehalose molecule in a motif A chain is relatively weakly bound and thus can easily “evaporate” back into the background sea of mobile trehalose molecules on the surface, as seen in the experiment (*cf.*Fig. 1 above).

**Fig. 5:**
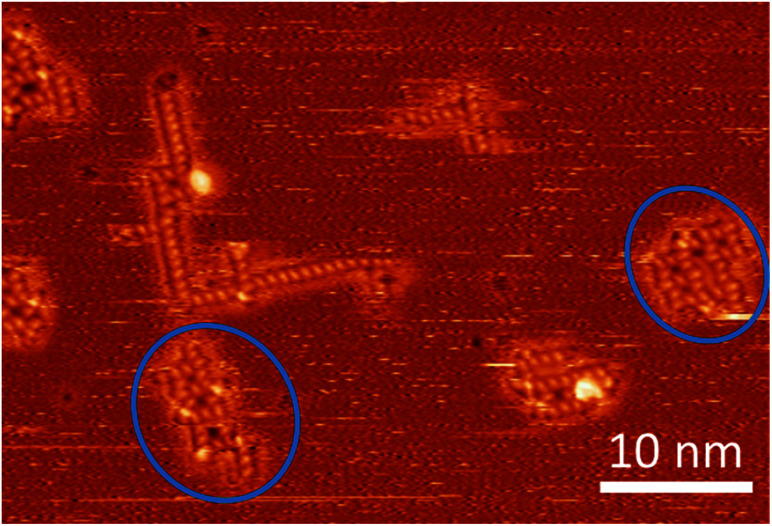
STM image at room temperature containing several assemblies of trehalose molecules, including two – rather strongly distorted – instances (marked with blue circles) where a second square pattern seems to be in the process to form adjacent to a motif B or one of its variations (*cf.* ESI†).

Restricting ourselves to motifs A and B, we note that motif A can easily be extended towards, in principle, infinite linear chains due to its commensurability with the substrate. In contrast, due to the more complex structure of motif B, one cannot construct an extended pattern by merging two motif B groups of molecules because placing two motif B groups side by side implies major distortions (see Fig. 5 and SI-6, as well as the discussion of motifs B and B′ and B′′ in the ESI†). On the other hand, the interaction of two adjacent parallel linear motif A is not very strong, either, since the ideal distance between two motif A chains (when ignoring the underlying Cu-lattice) is not compatible with the crystallographic repeat distance of the Cu-surface. Thus, there is no strong thermodynamic force driving the system to generate a dense coverage by motif A at finite temperatures.

Additionally, the observed polymorphism is closely related to the mobility of trehalose molecules on the surface because we observe fluctuations between motifs upon detachment and attachment of mobile molecules. Comparing Fig. 1 and SI-1,† we note the much higher density of individual molecules and small aggregates of trehalose molecules that are imaged on the cold substrate. This reflects the fact that at room temperature, a large fraction of the trehalose deposit is still mobile and thus only noticeable as streaks in the image. Nonetheless, the ability of trehalose to form stable clusters already at room temperature correlates with the fact that trehalose has one of the highest glass transition temperatures (*ca.* 393 K) of the disaccharides of biological interest.^24,25^ In contrast, sucrose (with a glass transition temperature of *ca.* 350 K) only forms stable aggregates far below room temperature.^17^ With respect to the time evolution of the trehalose aggregates at room temperature, three major mechanism should be considered: detachment–diffusion–attachment processes of individual molecules, analogous processes for small clusters of molecules, and shape fluctuations of larger clusters without break-up. While the last one will always be active to some extent, it cannot account for the large changes seen *e.g.* in Fig. 1. Regarding the first two mechanisms, mobility studies of atoms and atom clusters in two dimensions show that the diffusion coefficient *D* strongly depends on the diameter *R* of the cluster (*D* ∼ *R*^−4^).^26^ Thus, we expect the time evolution shown in Fig. SI-1† to occur *via* the evaporation of single trehalose molecules from the aggregate into the sea of mobile molecules followed by a re-attachment at another place on the evolving cluster, assisted by small fluctuations of the rim of the cluster.

From the above considerations, we conclude that, in the case of trehalose, a weakly diffusion-limited aggregation and evolution of the clusters is generating overall irregular shapes on the surface built up of several individual structural motifs.

We note that this polymorphism is rather different from the case of sucrose, in which we had observed self-organization of the sucrose molecules into an extended periodic arrangement only at low temperatures.^17^ Here, the optimal dense packing of the sucrose molecules on the Cu(100) surface produces a commensurable, four-molecule structure motif with *C*_4_-symmetry, which can be easily continued to infinity in both *x*- and *y*-direction with the same interaction strength in both directions, resulting in a homogeneous periodic structure. Furthermore, the sucrose motif does not compete with a strong alternative motif such as motif B in the case of trehalose. However, this periodic pattern was only observed at very low temperatures. At room temperature, no assemblies were visible in the experiment. The different thermodynamic stability of the assemblies might be related to the different numbers of intermolecular H-bonds per molecule in the patterns for trehalose (4–6 H-bonds) and sucrose (3 H-bonds), respectively: local re-arrangements towards the globally preferred structure are more easily achieved for sucrose due to its higher mobility on the surface in combination with the weaker molecular interactions (measured by the amount of H-bonds). At the same time, this means that the temperature at which permanent structures are established for sucrose molecules is lower than for trehalose.

The observed difference in the assembly motifs between trehalose and sucrose may also be related to the much higher efficiency of trehalose^27^ in the anhydrobiosis of biosystems, *i.e.* the protection of *e.g.* proteins, enzymes, peptides or lipid bilayers (*i.e.* membranes)^28–31^ against dehydration. While it is clear that the anhydrobiotic properties are also related to the nucleation and crystallization of the molecule, there are long-standing discussions concerning the general aspects and the precise details of the mechanisms how trehalose protects these biomolecular systems, such as vitrification or water entrapment.^1,5,25,32^

While we do not observe any glassy state of trehalose, which might be due to the low coverage, we find many holes of various sizes in the trehalose assemblies. These are reminiscent of the holes and channels observed in the trehalose + water simulations, which have been implicated in the remarkable anhydrobiotic properties of trehalose.^1^ The fact that we also observe such assemblies containing voids of various sizes already at room temperature on the time scale of STM measurements in a different environment, such as metal surfaces, indicates that open structures might intrinsically favoured by trehalose. Additionally, it supports the hypothesis that stable trehalose aggregates and channels can be crucial to the trapping of water around *e.g.* proteins, thus enhancing their protection against dehydration and freezing.

## Conclusions and perspectives

In summary, our STM-based investigations of trehalose molecules deposited *via* ES-IBD on a Cu-100 surface showed a large variety of assembly patterns both at room temperature and below liquid nitrogen temperature, which could be resolved as a combination of at least two basic compatible structural motifs, a linear chain of trehalose molecules of arbitrary length and a hollow square block made up of eight trehalose molecules. Using theoretical modeling we were able to reproduce the observed STM images and rationalize the appearance of the complex molecular aggregates. Although this study investigates trehalose on a metal surface, we note that already at room temperature we observe the same kind of structural elements, including empty regions inside the clusters, which have been found in earlier studies of trehalose–water and trehalose–biomolecule systems and have been suggested to play an important role in the anhydrobiotic properties of trehalose.

More generally, high-resolution imaging of surface assemblies enabled by ES-IBD can be used as an universal method to analyze the complex pattern formations of nonvolatile molecular species. By the combination of simulation and imaging, we can identify individual molecules on the surface and their conformations, making this method a very promising approach to characterize the structure of complex biomolecules and to understand their conformation at the submolecular level.

## Conflicts of interest

There are no conflicts to declare.

## Supplementary Material

RA-009-C9RA06764G-s001
