# Supplementary material for: Polymorphism in carbohydrate self-assembly at surfaces: STM imaging and theoretical modelling of trehalose on Cu(100)
Source: RSC Adv. 2019 Nov 4;9(61):35813–9. doi: 10.1039/c9ra06764g (PMC9074738; doi:10.1039/c9ra06764g)
Supplement: RA-009-C9RA06764G-s001 [file RA-009-C9RA06764G-s001.pdf]

Electronic Supplementary Material (ESI) for RSC Advances.
This journal is © The Royal Society of Chemistry 2019

Supporting information

Polymorphism in carbohydrate self-assembly at surfaces: STM imaging and theoretical modeling of trehalose on Cu(100)

Sabine Abb[†*], Nathalie Tarrat[‡], Juan Cortés[§], Bohdan Andriyevsky[¶], Ludger Harnau[#], J. Christian Schön[†], Stephan Rauschenbach[†], Klaus Kern[†$]

†Max Planck Institute for Solid State research, Heisenbergstr. 1, 70569 Stuttgart, Germany

‡CEMES, Université de Toulouse, CNRS, 29 rue Jeanne Marvig, 31055 Toulouse, France

§LAAS-CNRS, Université de Toulouse, CNRS, Toulouse, France

¶Koszalin University of Technology, Śniadeckich Str. 2, 75-453, Koszalin, Poland

#Bernhäuserstr 75. 70771 Leinfelden-Echterdingen, Germany

$Institut de Physique, Ecole Polytechnique Fédérale de Lausanne, 1015 Lausanne, Switzerland

Contents

General Procedure 2

1. Experimental methods 2

2. Theoretical methods 2

Additional experimental data 4

STM at low temperature 4

Further discussion 5

Discussion on the protonation and charge states of the trehalose molecule when deposited on the surface .5

From single molecule to assembly pattern 6

Discussion about the assembly patterns obtained using the different energy-minimum conformations for trehalose 7

Comparison of optimal conformations of a trehalose molecule in different environments 9

Motif B vs motif B' 12

Motif C: 16

General Procedure

1. Experimental methods

For electrospray-ion beam deposition (ES-IBD), the trehalose acquired from Alfa-Aesar (A19434) is dissolved in a water-ethanol 1:1 mixture to result in a concentration of 10^{-3} M. 0.1 % ammonia hydroxide was added to enhance ionization. The molecule remains stable in aqueous solution because no isomerism due to ring opening reactions is expected. This solution was used with our home-built ES-IBD to bring the molecule into the gas phase (2-3kV on the emitter, 20 µl/h flow rate, current up to -400 pA). Before deposition in UHV, the composition of the ion beam is monitored by a time-of-flight mass spectrometer, and the singly deprotonated disaccharide species was mass selected by a quadrupole to ensure a pure beam of trehalose ions (reduced current to 30%-50%). During the deposition (at a pressure of $6 \cdot 10^{-10}$ mbar), the molecular ion is decelerated to 5 eV to avoid fragmentation upon deposition. This high quality and purity of the samples is essential for reliable STM measurements. [1]

Prior to deposition, the Cu(100) single crystal (Mateck GmbH)) is cleaned by subsequent cycles of sputtering (1 kV, 30 min) and annealing (800 K, 10 min) and then transferred into the deposition chamber without breaking the vacuum. After deposition, the sample is transferred to the STM chamber into a variable temperature STM (Omicron VT-STM) where the measurements were conducted either at room temperature or at 40 K. Typical scan parameters are 91,6 pA and 1.87 V (with respect to the sample).

2. Theoretical methods

2.1 Global optimization

The global optimization of a single trehalose molecule on the Cu-100 surface was performed using the IGLOO algorithm (Iterative Global exploration and LOcal Optimization). The aim of IGLOO is not only to find the global minimum of an energy function, but to identify the major minima basins in the energy landscape. A key component of IGLOO is the rapidly exploring random tree (RRT) algorithm,[2] originally proposed for robot motion planning, but which has also been applied to model conformational transitions of peptides.[3,4] At each iteration of the IGLOO algorithm, multiple RRTs are constructed to globally explore regions of the energy landscape below a certain energy level, similarly to the threshold algorithm.[5] Configurations issued from the explorations are locally minimized and clustered. A representative configuration of each cluster becomes the root of a RRT tree in the next iteration of the IGLOO algorithm, and the whole process is repeated until a convergence criterion is satisfied.

The energy of the system was a sum of intramolecular terms, evaluated using the AMBER-GLYCAM potential,[6] and molecule-surface terms consisting of Lennard-Jones and electrostatic terms, where the latter were computed using the method of mirror charges.[7]

For computational efficiency reasons, we varied only the most important degrees of freedom during the global search: the location and orientation of the molecule on the surface (called the "pose"), and the twelve torsion angles of the trehalose molecule. The two rings were treated as rigid bodies at this stage; the conformations of the rings are rather stable at the temperatures of the experiment (RT and below[8, 9]), justifying this assumption. The choice of the initial chair conformations of the molecule was based on the models of the individual trehalose molecules in vacuum that had been generated using the GLYCAM Molecular Modelling Library (GMML).[6]

Because of a lack of information regarding the location of the hydrogen vacancy within the dehydrogenated trehalose molecules on the surface, we employed neutral molecules in the calculation instead of the singly deprotonated ions that are created during the ESI step. In gas phase studies, it has already been shown that the deprotonation site rapidly migrates and therefore delocalizes the charge of the molecule.[10] Preliminary investigations on surfaces showed that only minor changes in the overall conformations on the surface are induced by a single deprotonation, whatever the deprotonation site. Furthermore, the excess charge of the molecule is transferred to the metal substrate during absorption. Keeping in mind that the energy ranking of the different single molecule conformations might be different as function of the (de-)protonation state, we conducted our study by taking into account the three lowest-energy conformations issued from our global optimization scheme instead of the global minimum only (also see Further discussion: “Discussion on the protonation and charge states of the trehalose molecule when deposited on the surface“). For the Cu-100 surface, we used a perfect three-layer model, and the Cu-atoms were not allowed to move during the global IGLOO search. For more technical details of the IGLOO algorithm, we refer to reference[11] [22] in the main manuscript , and the associated SI, in particular.

2.2 Local refinement: DFT minimization

While experience has shown that the conformations do not change much during the subsequent ab initio local minimization, the ranking by energy can change. Thus, we consider the three minimum conformations with the lowest energies when performing the ab initio minimization, and during the construction of the multi-molecular models.

The ab initio minimization of the structure candidates placed on a slab of four Cu layers with the three lowest energies were performed on DFT level under periodic boundary conditions, with PAW pseudopotentials and the dispersion

corrected optB86b-vdw functional[12] implemented in the VASP code.[13-18] Interaction between the trehalose molecule and its periodic images was prevented by using large simulation cells: 5x5 unit cells in the x and y directions and at least 18 Å of vacuum between the trehalose molecule and the subsurface of the top box in the z direction. During the local optimization with a conjugate gradient algorithm, only the lowest Cu-layer was kept fixed - all other Cu atoms and all atoms of the trehalose molecule were allowed to move. In reciprocal space, only the Γ point was sampled, with a plane wave cut-off energy of 500 eV. The force convergence criterion was set to 0.01 eV/Å, and Methfessel-Paxton smearing was used with σ = 0.01 eV.[19]

2.3 Modeling of trehalose assemblies on Cu(100).

Generating accurate models of large molecular assemblies by a direct application of global optimization or MD simulation methods is extremely challenging. Instead, the models of the trehalose assemblies were constructed "by hand", using the three lowest-energy conformations of the single trehalose molecule on the Cu-100 surface as possible building blocks. We imposed constraints from the STM data on the model, such as relative orientations of the molecules, spatial dimensions of the observed features, and symmetries of the motifs and patterns, while ensuring that we kept the same orientation of each optimized trehalose molecule with respect to the underlying copper surface (also see Further discussion: "Discussion about the assembly patterns obtained using the different energy-minimum conformations for trehalose"). The simulated STM images were generated using the HIVE program. [20]

Additional experimental data

STM at low temperature

Upon cooling down the sample to 40K, the molecules that were mobile on the surface are now adsorbed and do not diffuse anymore as shown in Figure SI-1. Thus, the coverage on the surface appears much higher than at room temperature. Interestingly, the pure motifs are barely found on the surface, but an open network of a combination of all motifs A, B, and C is formed.

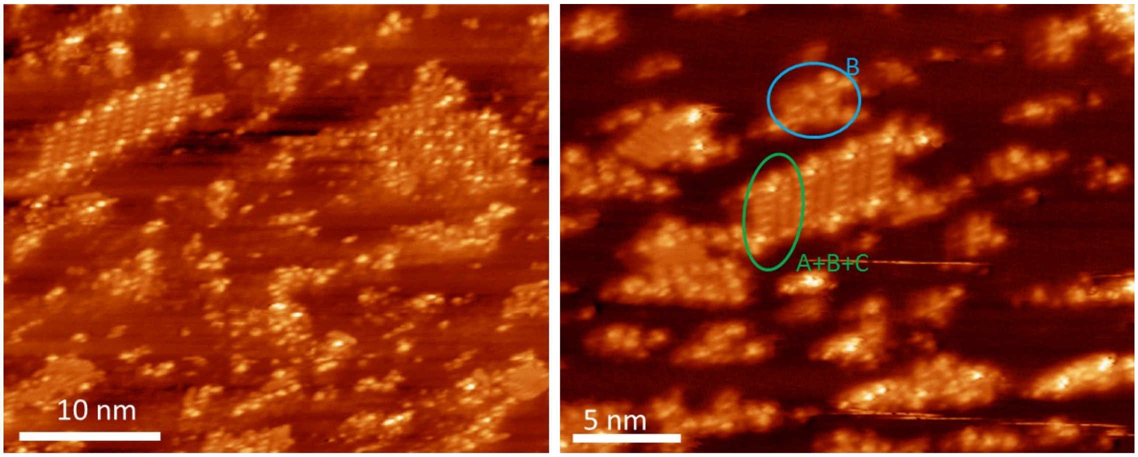

Figure SI-1. Low temperature STM images. Self-assembled open networks formed combining **motif A, B**, and **C** are observed on the surface at 40K.

Further discussion

Discussion on the protonation and charge states of the trehalose molecule when deposited on the surface

Concerning the state of the deprotonated trehalose molecule after deposition on the metal surface, we note that our calculations show that the excess negative charge of the deprotonated molecule is completely transferred to the metal substrate. Thus, after deposition, we can assume that we are dealing with a neutral dehydrogenated trehalose molecule on the surface in the experiment, and no longer with a deprotonated anion. This contrasts with the complete neutral molecule we employ in our global conformation searches and pattern modelling, due to the lack of information regarding the precise location of the hydrogen vacancy. Preliminary results of our ongoing theoretical work show that, firstly, the overall conformations of the complete and the dehydrogenated molecule are essentially the same, both in vacuum and on the surface, with only small deviations in the region of the molecule where the hydrogen atom is missing. Secondly, the additional covalent O-Cu (or possibly but unlikely C-Cu) bond strengthens the adsorption of the molecule to some degree compared with the neutral molecule, but does not strongly deform the conformation of the molecule nor cause a large change in its orientation with respect to the surface. Finally, even in vacuum, and much more on the metal surface, close-by H-atoms can migrate within the sugar molecule to the H-vacancy. Together with the fact that we have no information where the hydrogen atom was originally stripped from the trehalose molecule during the electrospray ionization in solution, this makes a precise assignment of the location of the H-vacancy practically impossible. Thus, we feel confident that employing the complete trehalose molecule in our computational modelling and searches is sufficient for the purpose of interpreting the experimental data.

From single molecule to assembly pattern

We have taken several precautions to ensure that it is reasonable to employ a conformation that had been optimized for a single trehalose molecule as the building block for the assembly patterns. First, we have observed that only small fluctuations in the conformation of a single molecule on a two-layer copper slab take place during long ab-initio molecular dynamics (AIMD) runs, as is exemplarily shown in the video in the SI entitled "Trehalose_Dynamics.xyz". This AIMD simulation has been performed using Langevin dynamics as implemented in the VASP package, together with PAW pseudopotentials and the dispersion corrected optB86b-vdw DFT functional.[12, 15-18, 21, 22] The trajectory length is 30ps, the temperature was set to 1000K, the time step to 1.5fs, and the damping coefficients to 2 ps^{-1} . This movie demonstrates that conformational changes are rare events, probably requiring a significant amount of activation energy and/or (simulation) time to occur. 200 structures were extracted from the AIMD trajectory and minimized. All the minima are in an energy range of 700 meV and no structural transition was observed during the minimization.

In addition, we have also investigated the conformational change of the molecule induced by the assembly process. To this end, we have formed two linear assemblies in two different orientations on the surface (orientation A along the [100] direction of the Cu(100) surface and orientation B along the [110] direction) of 5 trehalose molecules (**motif A**), which had been optimized as a single molecule conformation. DFT minimizations on the 5-trehalose patterns have been performed using VASP with PAW pseudopotentials and the optB86b-vdw functional, under periodic boundary conditions. A large simulation box was employed in order to avoid any spurious interaction between the disaccharide chain and its periodic images (x and y directions: 8 x 18 unit cells for the orientation A and 12 × 12 unit cells for the orientation B) and the subsurface (at least 18 Å of vacuum). Two copper layers were used to model the substrate (i.e. 576 Cu atoms). Both Cu-layers were kept fixed and all other atoms, i.e. the 5 trehalose molecules, were allowed to relax without constraints. Since the size of the supercell was large enough, the Brillouin zone sampling in reciprocal space restricted to the Γ point was sufficient to ensure a good convergence of the total energy. A plane-wave kinetic energy cut-off of 500 eV was employed and the convergence criterion was selected such that the maximum atomic force was less than 0.05 eV $Å^{-1}$. For dealing with the partial occupancies around the Fermi level, a Methfessel–Paxton smearing was used (with sigma = 0.01 eV). As seen in Fig. SI-2, the conformational changes before and after DFT optimization are very small. The only noticeable effect was an improvement of the H-bond network, demonstrating that no fundamental changes in adsorption position or conformation are expected when the individually optimized molecules are put together in an assembly at low temperatures. From these findings, we conclude that the multi-stage approach that we are employing to tackle the complexity of the investigated systems is reasonable and the models based on manually forming an assembly pattern of the single optimized molecules are justified.

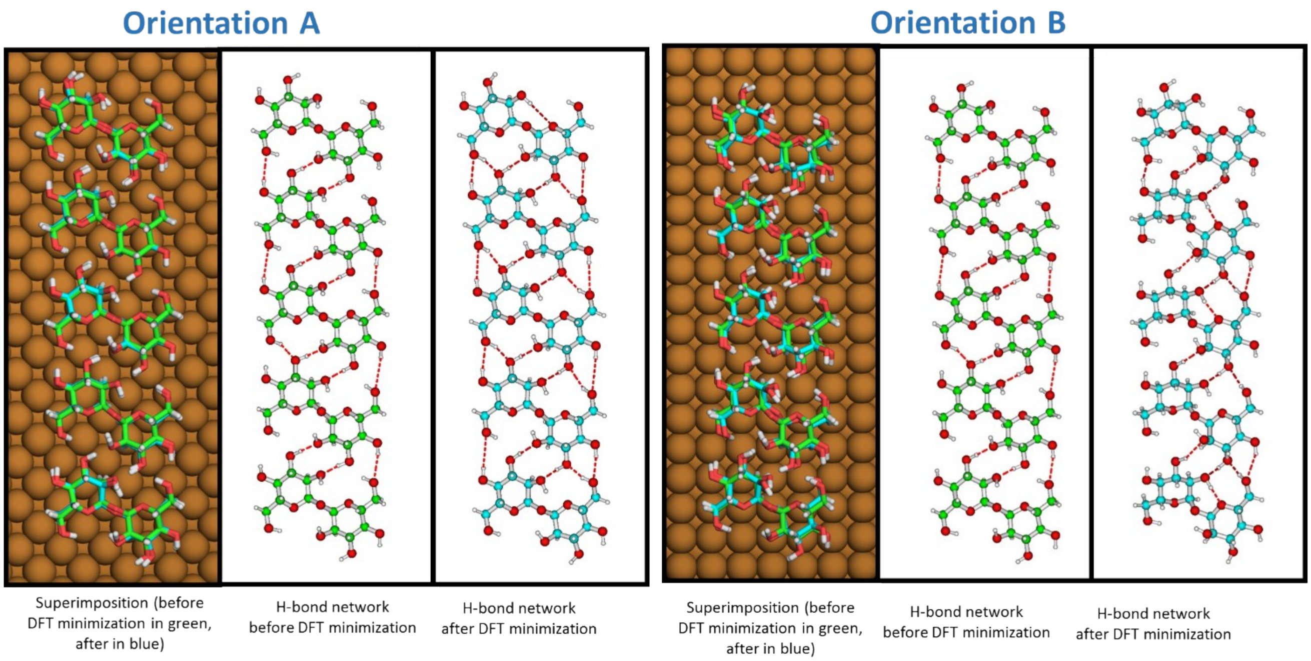

Figure SI-2. Conformational changes of trehalose molecules in an assembly for two different orientations. Green: before optimization, blue: after DFT optimization.

Discussion about the assembly patterns obtained using the different energy-minimum conformations for trehalose

The three DFT optimized energy-minimum conformations are employed to manually form models for **motif B**, which resemble the pattern in the STM image. It is important to note that the orientation of the molecule on the surface is kept in its optimized state, so that only constrained translations by the Cu-unit cell parameters as well as rotations of 90° and 180 ° are possible, reflecting the symmetries of the Cu-100 surface. The patterns were formed in such a way that the linear assembly of **motif A** was reproduced and then further put together to the quadratic octamer of **motif B**. Thus, we only show the possible patterns for **motif B**, as they already include the alignment of **motif A**.

A variety of different assembly patterns for **motif B** is given in Fig. SI-3. As can be seen in this figure, it is possible to form octamers with all three minimum conformations. However, when applying the constraints of the STM image, such as distance and symmetry, most of the obtained assembly patterns have to be excluded, as they are not necessarily 4-fold in symmetry when taking into account the surface lattice, or they exhibit overlapping molecules.

As none of the optimized conformations for the single trehalose molecule exhibit C2 symmetry, i.e., the two glucose units are not equivalent, there are two ways of building up the octamer structure of **motif B** differing only in the glucose unit that points to the center. The last row of Fig. SI-3 therefore shows a pattern in which the other glucose unit (in comparison to the first two rows) points to the center.

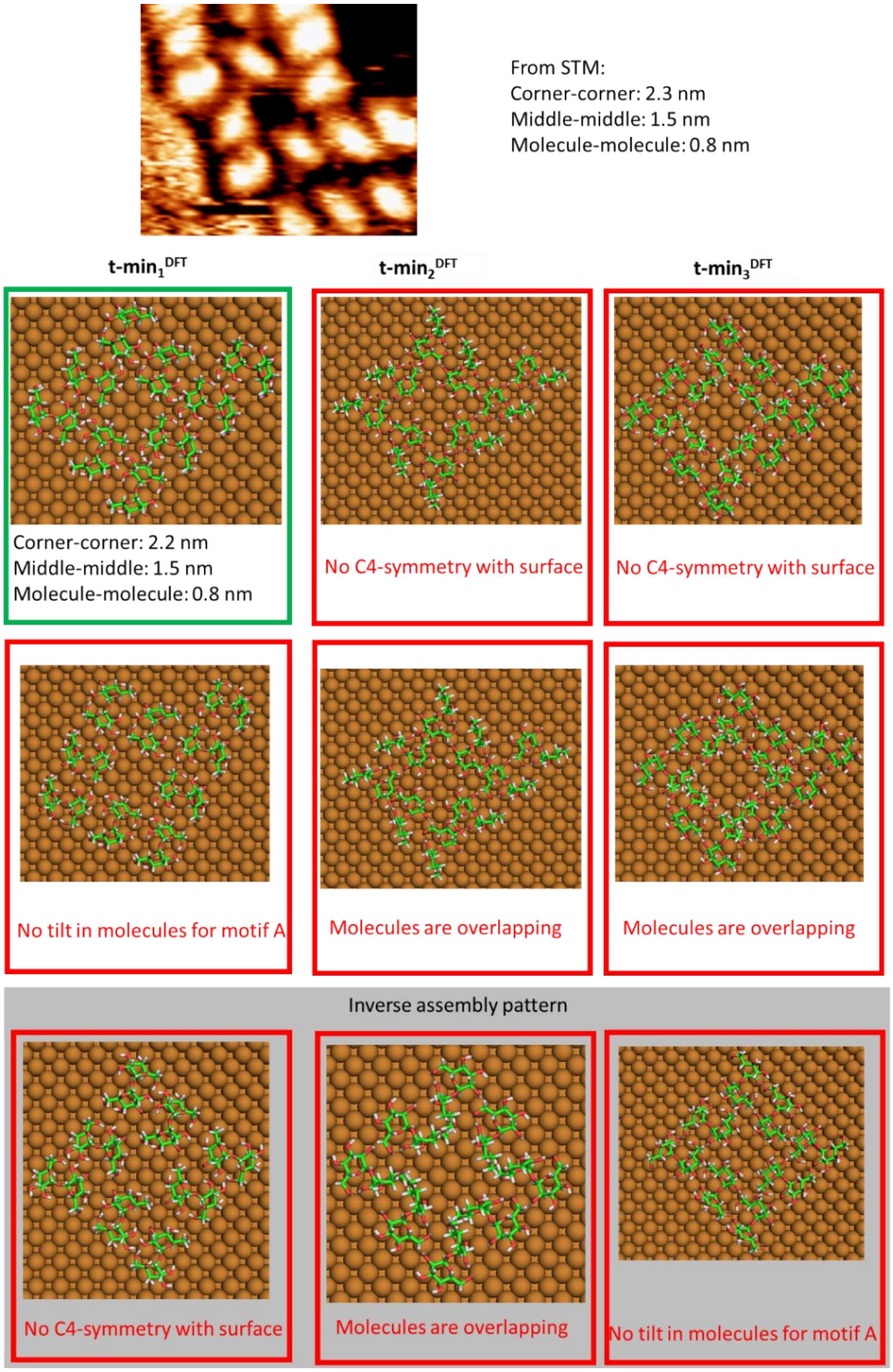

Fig. SI-3: Sample patterns that can be formed by just translation and rotation of the molecule with respect to the Cu(100) surface unit cell. Red frames mark the patterns that can be immediately excluded as they do not meet the STM data.

Only one assembly pattern is not rejected immediately, which is shown in Fig. SI-3. While most of the patterns fit to the dimensions of the measured STM data (spacing along the line: 0.9 nm, corner to corner: 2.3+-0.2 nm, and width of the assembly of 1.5 +- 0.2 nm), they are excluded by symmetry of the assembly on the surface (C4 symmetry with the surface) or the tilt of the molecules in the linear assembly of **motif A**. Thus, we have decided to take the assembly pattern of minimum t-min_1^{DFT} (marked with a green box) for all further discussions.

Comparison of optimal conformations of a trehalose molecule in different environments

One interesting question is to what extent the various conformations we have identified for the trehalose molecule on the surface match those found in crystalline anhydrous trehalose and those obtained from global optimizations in vacuum.

sample / angles	ψ_H	ϕ_H	ω	ω'	θ
Tre(h) [23]	-46.91	-59.54	69.83	-75.67	115.87
Tre(β) [24]	-57.43	-58.33	71.0	-72.1	113.3
Tre(α) [23]	-49.35	-65.26	-63.4	-61.0	113.0
t-min_1^{DFT}	-51.1	-23.1	51.4	63.5	112.4
t-min_2^{DFT}	-10.2	-29.0	57.1	59.1	114.2
t-min_3^{DFT}	-23.4	14.6	-176.1	103.1	116.2
t-min_1^{Vac}	-40.2	-37.5	58.8	59.9	113.1
t-min_2^{Vac}	18.7	-29.9	56.2	61.2	116.3
t-min_3^{Vac}	28.9	12.9	-179.7	60.2	115.4

Table SI-1: Comparison of trehalose conformations belonging to surface adsorbed minima (t-$min_{1,2,3}^{DFT}$), and those obtained from a global optimization in vacuum using the IGLOO method plus a subsequent DFT-optB86b-vdw refinement (t-$min_{1,2,3}^{Vac}$), with those of crystalline structures (Tre(α), Tre(β) and Tre(h)). Note that both t-min_2^{Vac} and t-min_3^{Vac} contain an intramolecular hydrogen bond. The energy differences of t-min_2^{Vac} and t-min_3^{Vac} with respect to t-min_1^{Vac} are about 12.6 kJ/mol and 12.9 kJ/mol, respectively.

ψ_H: HC1'- C1'-O1-C1; ϕ_H: HC1-C1-O1-C1'; ω: O5-C5-C6-O6; ω': O5'-C5'-C6'-O6'; θ: C1-O1-C1'. For numbering of atoms, c.f. figure SI-4.

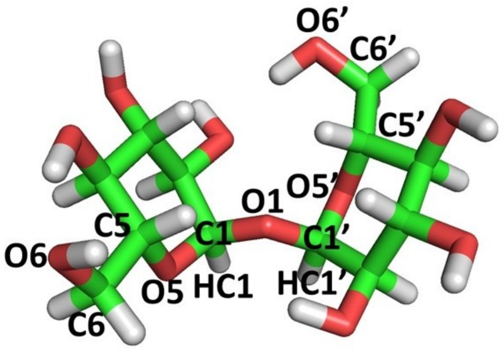

Figure SI-4: Trehalose molecule in stick format: green = carbon, red = oxygen, white = hydrogen. For clarity, only the atoms referred to in this study have been labeled.

Table SI-1 shows four characteristic dihedral angles (ψ_H, ϕ_H, ω, ω') that describe the overall conformation of a disaccharide, and the linkage angle along the glycosidic bridge (θ) for the three local minimum configurations of trehalose on the surface, three low energy minima found from global optimizations in vacuum, and those observed in the anhydrous crystalline modifications Tre(α) and Tre(β), and the hydrated modification Tre(h). We note that in all instances the angle θ along the bridge is very similar, while the dihedral angles ω and ω' can vary considerably. This is no surprise, since the atom groups associated with these angles are essentially free to move in the isolated molecule. Considering the location of these groups with respect to the surface, we find that they are close to the surface for t-min$_1^{DFT}$ and t-min$_2^{DFT}$ (in both cases less than 3 Å). In contrast, for t-min$_3^{DFT}$ one group points towards the surface, while the other one is located somewhat farther away.

Figure SI-5 shows the superpositions of the trehalose conformation taken from the Tre(α) crystal structure (black)[23] with the two minimum conformations t-min$_1^{DFT}$ (green) and t-min$_1^{Vac}$ (blue) on the surface and in vacuum, respectively. We observe that the experimental geometrical data (atom-atom distances, bond angles, puckering of the rings) are well reproduced by our calculations, validating the dispersion-corrected DFT calculations. However, for all three optimal conformations of the trehalose molecule on the surface and for all three found in vacuum, the relative orientation of the two rings is different from the one of the trehalose molecule in the crystalline environment, which is reflected in the dihedral angles ψ_H and ϕ_H. Indeed, the dihedral angles along the glycosidic bridge vary so that the disaccharide interacts in the most favourable way possible with its environment. Note that we know from our global energy landscape explorations of several disaccharides that, in vacuum,[5] there is not a very large energy barrier associated with these dihedral angles variations.

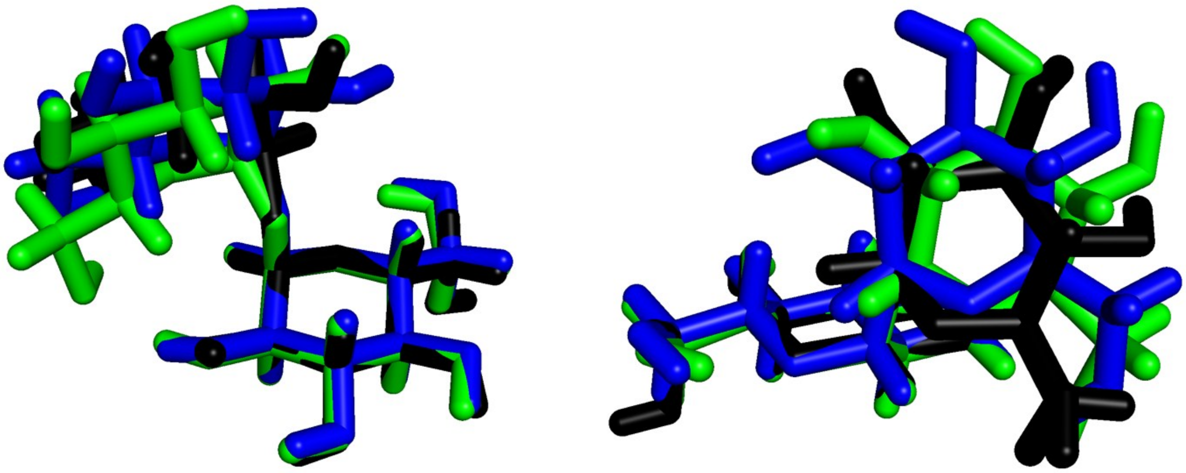

Figure SI-5: Superposition of the trehalose conformation taken from the Tre(α) crystal structure[23] [(black) with the lowest energy minimum conformation t-min$_1^{DFT}$ on the surface (green) and the lowest energy minimum conformation t-min$_1^{Vac}$ in vacuum (blue). The superposition was performed under the condition, to achieve optimal alignment of at least one of the two glucose rings. The left image shows the good alignment of the first glucose ring for all three structures, and the right image the difference in the location and orientation of the second glucose ring among the three conformations.

Motif B vs motif B'

Considering **motif B**, we note its rather complex arrangement of molecules. The motif itself is locally confined, but can extend by a combination of motif A and B (as described in the main manuscript). As figure SI-6a shows, employing the same building principle of pairs of trehalose molecules rotated by 90 degrees with respect to the next pair, but starting by using the lower wall of **motif B** as the upper wall of the next motif group, we achieve a related but different motif, which we call **motif B'**. As the larger interior space of the **motif B'** demonstrates, we are losing weak interactions (vdW and possibly H-bonds) between the trehalose molecules compared to the original **motif B**. And while glucose ring "A" was pointing outside everywhere in **motif B**, we do have two trehalose molecules in **motif B'** whose rings "A" point towards the interior of the motif. Overall, we are replacing two of the four ring-A'-ring-A' contacts in **motif B** by two ring-A-ring-A contacts, leading to two ring-A-ring-A and two ring-A'-ring-A' contacts in **motif B'**. On a qualitative level, we would expect that the more compact structure and the presence of four identical glucose-ring contacts in **motif B** will favor **motif B** over **motif B'**, but a definitive answer will require further ab initio calculations of the different motifs and their possible connectivities. In the experiment, we find a variety of very similar structures (see Fig. SI-6 b and c) that are formed by multiple motif B variations joined together, such as via a joint corner or via a joint side, as shown in Fig. SI-6. The respective building principle is represented in the cartoon inf Fig. SI-6 d and e.

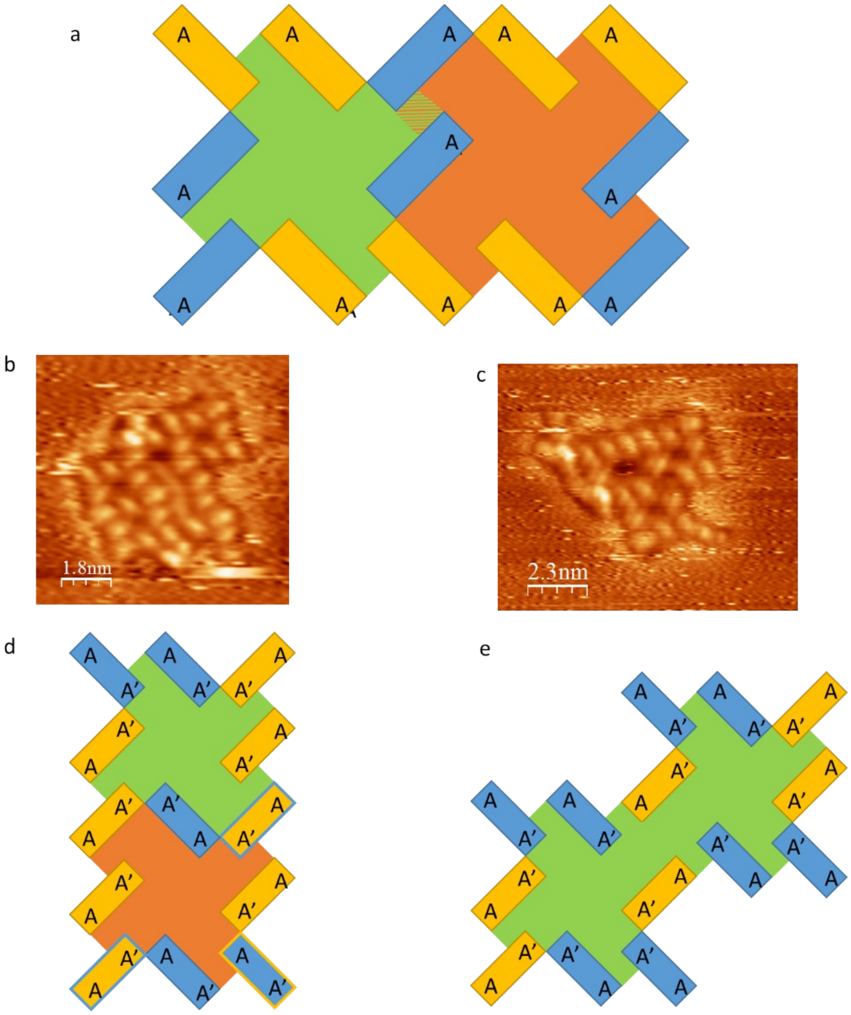

Figure SI-6: a) and b) STM image of joint motif B and its variations. c) Sketch of **motifs B** and **B'**, consisting of four pairs of trehalose molecules each arranged in a square-walled pattern. Yellow and blue rectangles indicate the individual trehalose molecule units, with A (and A') indicating always the same glucose subunit. Note that in **motif B** (square on the upper part of a)), all A rings point outwards, and in **motif B'** (square on lower part), two point towards the inside. Clearly, the interior of the **motif B'** is larger than the one of **motif B** (indicated by the orange and the green area, respectively, inside the motifs), most likely leading to the loss of favorable interactions between the molecules.

We suspect that the automatic appearance of variations of **motif B**, such as **motif B'**, is caused by the difficulty to extend **motif B** towards infinity: one cannot use the "right" wall of one **motif B** group as the "left" wall of a second **motif "B"** group - the resulting **motif B'** we could construct in this fashion would likely be unfavorable

energetically. Similarly, placing two "right" walls of **motif B** next to each other (an arrangement actually favored by the **motif A** !) does not work, since we again would be forced to generate a **motif B'**. But if we set two **motif B** groups next to each other such that the molecules of a "right" wall are adjacent to the molecules of a "left" wall, this does not correspond to an energetically favorable trehalose-trehalose neighbor arrangement, since it now implies, like in **motif B'**, the existence of ring-A-ring-A junctions (see figure SI-7).

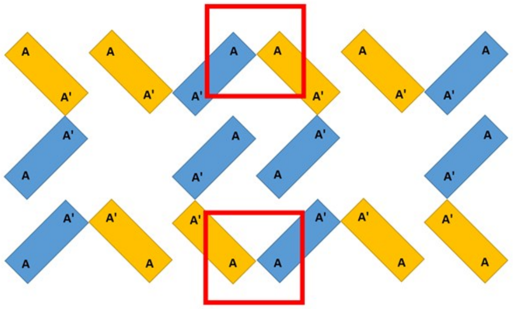

Figure SI-7: Sketch of two adjacent **motifs B**. The (unfavorable) ring-A-ring-A contacts are marked in red.

Note that because of the opposite orientations of the trehalose molecules at opposite corners, we cannot continue the **motif B** via identifying opposite corners, either. As a consequence, a long-range extension of **motif B** has to either involve **motif B'** (including distortions) or requires us to place two **motif B** groups wall-to-wall to each other, producing an unfavorable arrangement of neighboring molecules. Of course, if the interaction of the molecules is not affected by the different orientations of the molecules in **motifs B** and **B'** and neither by the larger interior distances, then such a **B-B'-B** pattern is feasible, in principle.

Nevertheless, the STM images in figure SI-6 demonstrate that assemblies containing adjacent **motifs B/B'** are observed in the experiment. However, we note that the motifs become rather strongly distorted in the attempt to match edges of the two **motifs B/B'** while keeping the energy of the resulting configuration to a minimum, supporting our expectation that **motif B'** is not as favorable energetically as **motif B**.

Clearly, the relative energies of the various motifs, including **motif A**, of course, and the details of the interactions of the trehalose molecules making up the motifs, where both the interactions between the trehalose molecules and those between the molecules and the surface need to be considered, are important open questions. Their investigation are ongoing topics of research in our group, but would go beyond the purview of this study;

preliminary results indicate that the energy differences are probably comparable with the thermal energy in the system during the pattern formation.

Motif C:

An open question regarding the assembly pattern is the observation of **motif C** in the STM images. The line profile through this motif in Fig. SI-8 shows exemplarily the height of the assembly: While the motifs discussed in the main text formed by trehalose molecules adsorbed on the surface show a height up to 1.5 A, the central feature exhibits a height of ~2.7 A. Moreover, this central feature consists of two very bright very close-by (nearly merged) spots, which together encompass about the area of single trehalose molecule. These observations suggest that the intense central feature also corresponds to a molecule. The unusally extended height raises the possibility that the molecule is slightly raised from the surface or might even be adsorbed on the first trehalose layer, presenting a strong intermolecular interaction. Here, the issue is the stability of such an arrangement, especially since we do not observe a dense packing of trehalose molecules in the area around the bright spot which could serve as a pedestal for the raised molecule. Moreover, it is possible that this molecule is adsorbed in another conformation, similar to the bowl-like conformation of t-min3DFT, which would account for two observed features as presented in Fig. SI-8. However, we lack experimental or theoretical information regarding which conformation would be the preferred one when placed on top of a layer of trehalose molecules as the conformation might be totally diferent due to the underlying molecules instead of a metal substrate.

A related alternative suggestion is that we might be seeing two neighboring trehalose molecules rotated by 90 degrees out of the plane - in that case, the molecules would extend considerably higher than the surrounding trehalose molecules explaining the very bright large (double) spot in the STM-images. Furthermore, the attraction between the two molecules might be strong enough to stabilize the formation, preventing the molecules to fall back onto the surface into one of the other minimum conformations, especially if they are surrrounded by a cluster of additional trehalose molecules. While each of these suggestions has some plausible arguments in its favor, a definitive answer would require additional extensive experimental and theoretical investigations, which would go beyond the purview of the current study.

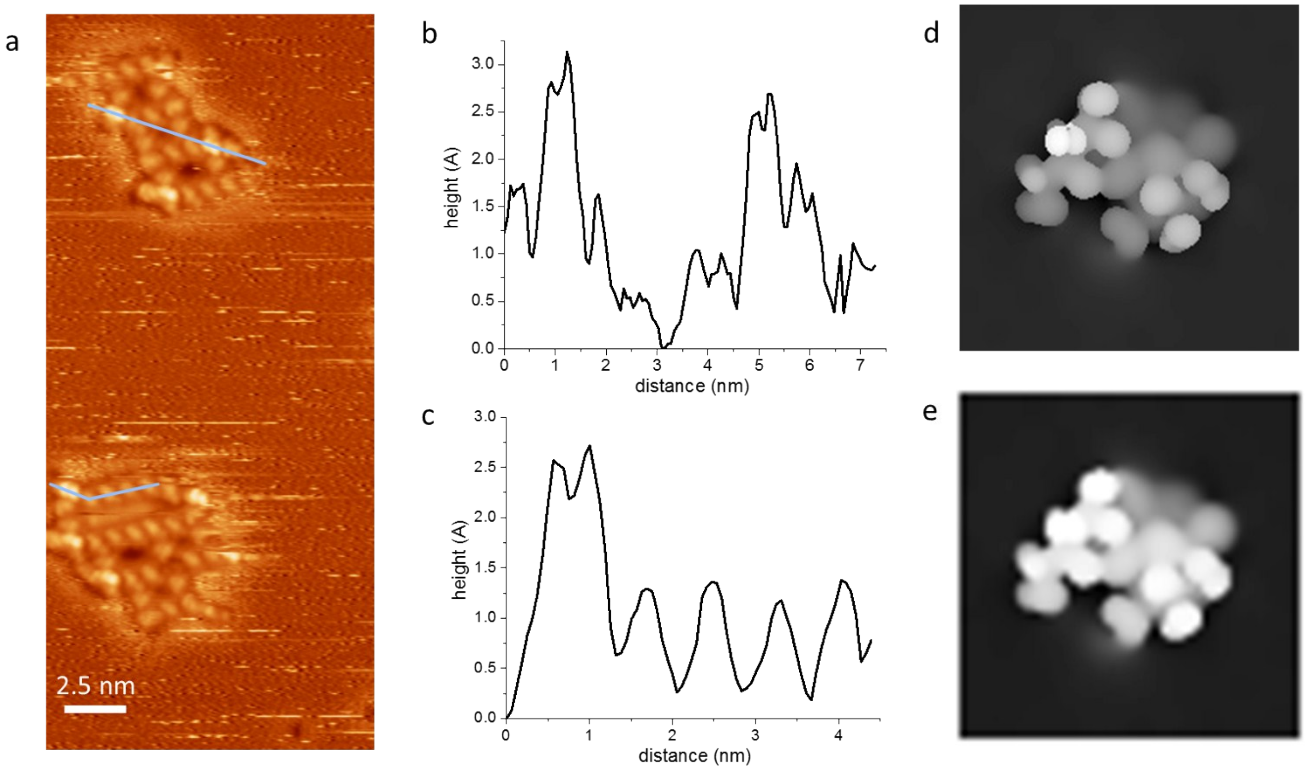

Figure SI-8: Characterization of motif C. a) STM image at room temperature presenting several motif C structeres in the assembly. b) and c) Corresponding line profiles to the lines in the STM image. d) and e) simulated STM images of t-min$_3^{DFT}$ with different sample-tip distances.

References

1. S. Rauschenbach, M. Ternes, L. Harnau and K. Kern, *Annu. Rev. Anal. Chem.*, 2016, **9**, 473-498.
2. S. M. LaValle, *Planning Algorithms*, Cambridge University Press, 2006.
3. L. Jaillet, F. J. Corcho, J.-J. Pérez and J. Cortés, *J. Comput. Chem.*, 2011, **32**, 3464-3474.
4. D. Devaurs, K. Molloy, M. Vaisset, A. Shehu, T. Siméon and J. Cortés*, *IEEE Trans. NanoBiosci.*, 2015, **14**, 545-552.
5. S. Neelamraju, C. Oligschleger and J. C. Schön, *J. Chem. Phys.*, 2017, **147**, 152713.
6. K. N. Kirschner, A. B. Yongye, S. M. Tschampel, J. González-Outeiriño, C. R. Daniels, B. L. Foley and R. J. Woods, *J. Comput. Chem.*, 2008, **29**, 622-655.
7. J. D. Jackson, *Classical Electrodynamics*, Wiley, 2012.
8. Z. Kan, X. Yan and J. Ma, *J. Phys. Chem. A*, 2015, **119**, 1573-1589.
9. J. Xia and D. A. Case, *Biopolymers*, 2012, **97**, 276-288.
10. W. B. Struwe, C. Baldauf, J. Hofmann, P. M. Rudd and K. Pagel, *Chem. Commun.*, 2016, **52**, 12353-12356.
11. S. Abb, N. Tarrat, J. Cortés, B. Andriyevsky, L. Harnau, J. C. Schön, S. Rauschenbach and K. Kern, *Angewandte Chemie*, 2019, **131**, 8424-8428.
12. M. Dion, H. Rydberg, E. Schröder, D. C. Langreth and B. I. Lundqvist, *Phys. Rev. Lett.*, 2004, **92**, 246401.
13. G. Kresse and J. Hafner, *Physical Review B*, 1993, **47**, 558-561.
14. G. Kresse and J. Hafner, *Physical Review B*, 1994, **49**, 14251-14269.
15. G. Kresse and J. Furthmüller, *Computational Materials Science*, 1996, **6**, 15-50.
16. G. Kresse and J. Furthmüller, *Phys. Rev. B*, 1996, **54**, 11169-11186.
17. P. E. Blöchl, *Phys. Rev. B*, 1994, **50**, 17953-17979.
18. G. Kresse and D. Joubert, *Phys. Rev. B*, 1999, **59**, 1758-1775.
19. M. Methfessel and A. T. Paxton, *Phys. Rev. B*, 1989, **40**, 3616-3621.
20. D. E. P. Vanpoucke and G. Brocks, *Phys. Rev. B*, 2008, **77**, 241308.
21. G. Kresse and J. Hafner, *Phys. Rev. B*, 1993, **47**, 558-561.
22. G. Kresse and J. Hafner, *Phys. Rev. B*, 1994, **49**, 14251-14269.
23. H. Nagase, N. Ogawa, T. Endo, M. Shiro, H. Ueda and M. Sakurai, *J. Phys. Chem. B*, 2008, **112**, 9105-9111.
24. G. A. Jeffrey and R. Nanni, *Carbohydr. Res.*, 1985, **137**, 21-30.
